# AGING ALTERS STEROID HORMONE METABOLISM AND EXACERBATES LOWER URINARY TRACT DYSFUNCTION IN MICE

**DOI:** 10.1093/geroni/igz038.3248

**Published:** 2019-11-08

**Authors:** Teresa T Liu, William A Ricke

**Affiliations:** University of Wisconsin - Madison, Madison, Wisconsin, United States

## Abstract

Benign prostatic hyperplasia (BPH) is a disease of aging that impacts 50% of men in their 50s and 90% of men in their 80s. While rodent models have been invaluable in the study of lower urinary tract dysfunction (LUTD) associated with human disease, many studies recapitulate aspects of aging, steroid hormone fluctuations, and/or inflammation without using aged mice that would better correspond to the age range of patients. In this study, we examine the impact of age in the hormone-induced mouse model of LUTD, so we can better understand the contribution of age to disease initiation and progression. We’ve discovered that aged mice exhibit a level of LUTD that is further exacerbated by hormone implantation when compared to both treated and untreated younger mice. Examination of circulating levels of androgens and estrogens indicate an alteration in steroid hormone metabolism with age, suggesting an altered nuclear receptor activation within disease. Epigenetic modifications have been associated with normal aging, including an increase in DNA methylation to alter gene expression. Examination of the proximal promoter of a steroid enzyme gene, CYP7B1, responsible for the degradation of 3β-diol (an ERβ ligand) within the prostate shows an age-mediated increase in methylation. With this, there is a corresponding decrease in CYP7B1 gene expression in the aged mice. Taken together, this suggests the altered steroid hormone environment seen in LUTD is further exacerbated due to normal aging-related epigenetic modifications that need to be considered for better treatment efficacy.

